# Outpatient penicillin allergy de-labeling in primary care—whom to target

**DOI:** 10.1017/ash.2026.10331

**Published:** 2026-03-27

**Authors:** Angie Ton, Dan Ilges

**Affiliations:** Mayo Clinic Arizona, USA

## Abstract

Education and allergy referral via nurse-driven online messaging to patients (n = 1,284) with penicillin allergy labels (PALs) reduced PALs by 8.6%. The percentage decrease of “unknown” (14.8%) and “rash” (7.9%) PALs were higher vs “hives” (6.1%) and “itching” (1.4%) reaction types.

## Introduction

The majority of penicillin allergy labels (PALs) are inaccurate, with estimates of <1 of 10 adults having a true allergy.^1^ Inaccurate PALs unnecessarily limit antibiotic selection and can result in less effective, less safe, and unnecessarily broader-spectrum antibiotics, increasing antibiotic resistance and costs to the patient and health care system.^1,2^ PALs are especially barriers to care when patients are unable to provide further clarification during acute illness and/or are uninterested in penicillin allergy challenge when they are already feeling unwell. Therefore, addressing PALs in the outpatient setting can help facilitate appropriate antibiotic selection before patients find themselves in need of emergency or inpatient care. We previously described the utility of nurse-driven direct messaging to primary care patients with unspecified PALs, which provided education, offered penicillin allergy testing via allergy referral, and requested clarification on the reaction type, resulting in a 45.4% reduction in unspecified PALs and 19.9% removal of PALs.^3^ Since the initial pilot project, this effort was expanded to include a second primary care clinic and additionally included specified allergy labels of rash, itching, and hives. This project aims to identify the utility in expanding messaging to patients with these specific penicillin allergies and which reactions to prioritize de-labeling in the outpatient space.

## Methods

This study was conducted at the Mayo Clinic in Arizona as part of routine quality improvement. Therefore, the need for institutional review board approval was waived. Electronic medical record query tools were utilized to identify adult patients with PALs at the Mayo Clinic Family Medicine primary care sites in Chandler and Glendale Arizona, referred to as San Tan and Arrowhead, respectively. All penicillins and derivatives were included in the query included as previously described.^3^ A total of 2,118 patients were identified with a PAL out of 10,606 empaneled patients, or about 1 in 5. Due to the volume of patients identified, patients were sub-grouped by allergic reaction listed. A previous project demonstrated the utility of messaging patients with an unspecified PAL to clarify their allergy and offer penicillin allergy testing.^3^ This project aimed to identify if there is utility in expanding messaging to patients with specified PALs and which reactions to target. Due to the subjective nature of and variability in severity of patient-reported reactions of rash, hives, and itching, the project was expanded to include these 3 reactions. A message was created (see supplemental material) providing patient education regarding the common misdiagnosis of penicillin allergies and requests the patient to (1) specify their reaction and/or (2) inquire if they are interested in an allergy consult for penicillin allergy testing. The message provided this education in written format, as well as through an educational video.^4^ A team of 5 nurses sent these messages to an average of 10 patients per day exclusively through their online patient portal over the month of February 2024. Protocols allowed nurses to place outpatient allergy consult referral and update or add details to patient’s PAL listed in the electronic medical record. Physician support was available to nursing when needed. Data collection was completed six months status post intervention.

## Results

**Figure 1.**
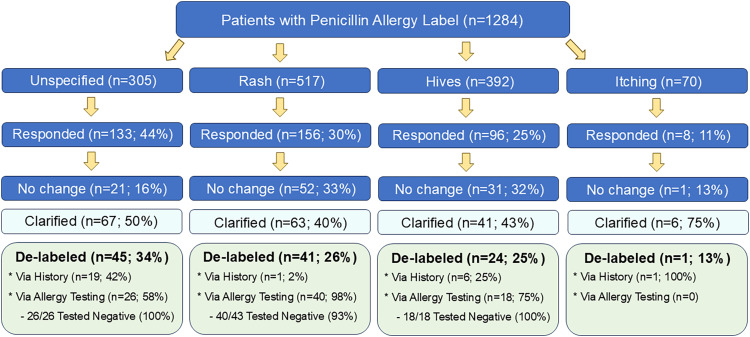
Results of intervention for patients with penicillin allergy labels.

Of the 1,284 patients identified and messaged, 110 (8.6%) patients had their PAL removed. Among 305 patients with an unknown PAL, 45 patients were de-labeled, for a 14.8% reduction. Of the 26 patients who completed allergy testing, all tested negative (100%). Among 517 patients with a reported history of rash, 41 patients were de-labeled, for a 7.9% reduction. Of the 43 patients who completed allergy testing, 40 tested negative (93%). Among 392 patients with a history of hives, 24 patients were de-labeled, for a 6.1% reduction. Of the 18 patients who completed allergy testing, all tested negative (100%). Among the 70 patients with a history of itching, 1 patient was de-labeled for a 1.4% reduction. None completed allergy testing. [Table 1, Figure 1]. Of the patients who completed penicillin allergy testing, a total of 96.6% (84/87) were negative. Of the patients who were de-labeled through history, reasons included remote history of allergy testing at outside institutions but failure to update their PAL, incorrect antibiotic listed, patient did not personally recall experiencing, and consented to removal. Chi-square analysis showed there was no statistically significant difference in outcome between the San Tan versus Arrowhead clinic (*P* = .708) and that the difference in reduction amongst the reactions is not due to chance (*P* < .001).

**Table 1.** Percent reduction of penicillin allergy labels by reaction and clinic post intervention

Reaction type	Pre	Post	% Reduction	n Reduction
Unknown	305	260	14.8	45
Rash	517	476	7.9	41
Hives	392	368	6.1	24
Itching	70	69	1.4	1
Clinic	Pre	Post	% Reduction	n Reduction
San Tan	450	403	10.4	47
Arrowhead	834	771	7.6	63
**Total**	1,284	1,174	8.6	**110**

## Discussion

PALs are widely reported but frequently inaccurate and are associated with worse health outcomes. While de-labeling inaccurate PALs represents an important opportunity to improve health outcomes on both an individual and a population level, the work required to address the sheer volume of PALs, which are reported in ∼10% of Americans, is immense.^1^ Our cohort of empaneled primary care patients revealed an even higher rate of PALs at ∼20%. Confounding this issue further, especially in the United States, is limited access to specialist and even primary care, particularly in rural communities.^5^ Thus, creative approaches to optimally and safely reduce PALs that utilize existing resources and workflows while limiting strain on primary care are desperately needed.

One such approach that we’ve described herein is nurse-driven direct-to-patient messaging with education and optional allergy referral. We found this approach to be an effective method of reducing inaccurate PALs in the outpatient setting. Our intervention was most clinically significant for our patients with PALs that were unknown, followed by patients with PALs listed as rash, then hives, and itching. Of the patients who completed allergy testing in our cohort, 96.6% (84/87) were negative. This is in-line with other de-labeling projects conducted in the obstetric, surgical, oncology, pediatric, military, and inpatient settings, which achieved 86%–99% reduction in PALs.^6^ Moving forward, we will focus our finite resources on the top two PALs that demonstrated the highest yield PAL reduction, which were unknown and rash, at our institution’s other primary care sites.

Regarding specific allergy types, “unknown” reactions most likely occurred in childhood or remote past, if at all. Reactions that occurred greater than 5 years ago without memorable reaction or treatment are considered low risk for true allergy.^7^ The majority of rashes are more likely due to infection-related exanthem than a true allergy.^8^ In our cohort, hives and itching had the lowest response rates and thus the lowest rates of de-labeling. We hypothesize that the perceived severity (eg hives) and the association with a specific negative symptom (eg itching) may have contributed to the lower response rates in these populations; however, additional studies on patient perceptions of allergies are needed.

This study has several limitations that may limit generalizability to other institutions or practices, including access to in-network allergy referral and a patient population with high utilization of out online patient messaging platform, which may have influenced our response and referral acceptance rates, respectively.

In conclusion, our nurse-driven protocol helped to specify and reduce PALs in the outpatient setting prior to acute illness, facilitating increased antibiotic options for when patients require emergency or inpatient care. Future directions include exploring the utility of using this protocol to address other PALs that may represent adverse effects rather than true allergies (eg diarrhea or vomiting) to facilitate direct de-labeling. This protocol might also be adapted and applied in other health care systems with direct patient messaging platforms.

## Supporting information

10.1017/ash.2026.10331.sm001 Ton and Ilges supplementary material Ton and Ilges supplementary material

## Supplementary material

The supplementary material for this article can be found at https://doi.org/10.1017/ash.2026.10331.
