# Supplementary figures and images for: Outpatient penicillin allergy de-labeling in primary care—whom to target
Source: Antimicrob Steward Healthc Epidemiol. 2026 Mar 27;6(1):e73. doi: 10.1017/ash.2026.10331 (PMC13104558; doi:10.1017/ash.2026.10331)

SUPPLEMENTAL MATERIALS

Message sent to patients:


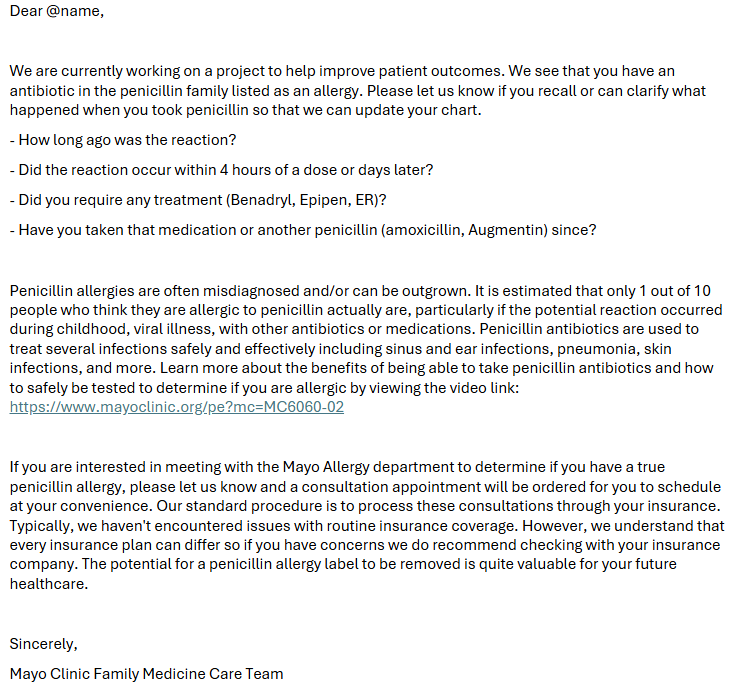

Supplement: Ton and Ilges supplementary material [file S2732494X26103313sup001.docx]
